# Acknowledgement to Reviewers of *Journal of Developmental Biology* in 2018

**DOI:** 10.3390/jdb7010002

**Published:** 2019-01-09

**Authors:** Journal of Developmental Biology Editorial Office

**Affiliations:** MDPI, St. Alban-Anlage 66, 4052 Basel, Switzerland

Rigorous peer-review is the corner-stone of high-quality academic publishing. The editorial team greatly appreciates the reviewers who contributed their knowledge and expertise to the journal’s editorial process over the past 12 months. In 2018, a total of 30 papers were published in the journal, with a median time to first decision of 13 days and a median time to publication of 34.5 days. The editors would like to express their sincere gratitude to the following reviewers for their cooperation and dedication in 2018:

Ahringer, JulieLawrence, ChristianAllen, ClaireLinker, ClaudiaArtinger, KristinLiu, AiminAskjaer, PeterLiu, KarenAtukorallaya, DeviLupo, GiuseppeBaburamani, Ana A.Miller, GalenBijlsma, Maarten F.Mishina, YujiBlaess, SandraMukhopadhyay, SaikatBohnsack, Brenda L.Muñoz-Chápuli, RamónBowling, HeatherMurray, MichaelCandiani, SimonaMuto, AkiraCao, XinweiNiswander, LeeCerveny, KaraNohno, TsutomuChin-Sang, IanOhba, ShinsukeClavel, Marie-AnnickPacifici, MaurizioCray, James JohnPadian, KevinCrompton, TessaPal, KasturiDe La Cova, ClairePan, YongchuDe Navascues, JoaquinPeuhu, EmiliaDettman, RobertPowder, KaraEkker, MarcRelaix, FredericFernández, IgnacioRiobo-Del Galdo, NataliaFink, Rainer H. A.Rocheleau, Christian E.Finnell, RichardRöper, KatjaFranz-Odendaal, TamaraRousset, RaphaëlGantz, StephanieSánchez-Camacho Blázquez, CristinaGassmann, RetoSchafer, BillGeisbrecht, Erika R.Schneider-Maunoury, SylvieGiangrande, AngelaSchwartz, Lawrence M.Giniger, Edward S.Seeger, MarkGoyal, YogeshShlizerman, EliGrobe, KayShuler, CharlesGroßhans, JörgSi, KausikHall, David H.Sripathy, SmithaHerránz, HéctorSztal, TamarHilgers, ValérieThor, StefanHufnagel, RobertThyme, SummerIwata, JunichiTien, Lu-TaiJohnston, ChristopherVora, MehulKanczler, JanosWang, Bang-AnKappen, ClaudiaWang, Wen-DerKerosuo, LauraWeake, Vikki M.Khoshmanesh, KhashayarWeatherbee, Scott D.Korona, DagmaraWolman, Marc A.Köster, Reinhard W.Yelick, PamelaKwan, KristenZaffran, StéphaneLacin, Haluk


